# A Retrospective Analysis of Dynamic Compression Plating Versus Intramedullary Nailing for the Management of Shaft of Humerus Fractures in an Urban Trauma Care Center

**DOI:** 10.7759/cureus.52883

**Published:** 2024-01-24

**Authors:** Dhruva Angachekar, Shivam Patel, Shaswat Shetty, Shubham Atal, Amit Dhond, Raunak Sharma, Pranav Nagargoje, Dhairya Angachekar

**Affiliations:** 1 Orthopaedic Surgery, Paramount Hospital and ICCU, Mumbai, IND; 2 Orthopaedics, P. D. Hinduja Hospital and Medical Research Centre, Mumbai, IND; 3 Orthopaedics and Traumatology, Lokmanya Tilak Municipal Medical College and General Hospital, Mumbai, IND; 4 Trauma and Orthopaedics, Bharat Ratna Dr Babasaheb Ambedkar Municipal General Hospital, Mumbai, IND; 5 Orthopaedics, Bharat Ratna Dr Babasaheb Ambedkar Municipal General Hospital, Mumbai, IND; 6 Medical Education, MGM Medical College and Hospital, Navi Mumbai, IND

**Keywords:** trauma management, upper extremity trauma, intramedullary locked nail, dynamic compression plating, humerus shaft fractures

## Abstract

Introduction

There is constant debate regarding the best surgical technique for the fixation of shaft humerus fractures. Intramedullary nailing and dynamic compression plating are the most popular surgical options.

Materials and methods

In our study, we retrospectively analyze the results of 27 patients with shaft humerus fractures managed with intramedullary nailing (10) and dynamic compression plating (17) at our institute from September 2021 to October 2022. Preoperative clinical assessment sheets, postoperative follow-up sheets, operative notes, anesthesia sheets, and preoperative and follow-up radiographs were analyzed. Reamed antegrade nailing was done in all cases, while dynamic compression plating was done through a posterior approach.

Results

The operative time of the nailing group was 82.1 ± 7.61 mins, which was significantly lesser (P value <0.05) than that of the plating group, which was 119.59 ± 10.16 mins. The intraoperative blood loss of the patients who were managed with nailing was 71 ± 7.38 mL, which was significantly lesser (P value <0.05) than that of the plating group, which was 130.59 ± 11.44 mL. The patients in both groups had a statistically nonsignificant difference in terms of functional results, which were assessed using Rodriguez-Merchan criteria. Complications were similar in both groups with infection (17.65%), and postoperative radial nerve palsy (11.76%) was more common among the patients undergoing plating, and shoulder impingement(20%) was common among those undergoing nailing.

Conclusion

This study concluded that both surgical options are similar in the case of functional results. The selection of the surgical method should be as per the surgeon's surgical familiarity and personalized to individual patients.

## Introduction

About 1-3% of all fractures encountered in orthopedic practice are humeral shaft fractures [1]. Around 90% of simple humeral shaft fractures are predicted to heal without any form of surgical intervention. The techniques include the shoulder spica cast, functional brace, hanging cast, and Velpeau dressing [2]. Despite this, there are some circumstances wherein primary or secondary surgical treatment is indicated [3]. Operative intervention lowers the possibility of nonunion and could produce improved functional outcomes. To treat these fractures, surgical intervention is being used more frequently nowadays. The best surgical method, however, is still up for discussion. The two surgical techniques that are most frequently employed are open reduction with plate fixation and nailing [4,5]. Utilizing a dynamic compression plate necessitates a lengthy procedure that involves extensive dissection of soft tissues from the bone and problems because the radial nerve is close by in the field of dissection [6]. It is theoretically possible to stabilize the fracture using the intramedullary nailing with less invasive surgery, with a biomechanical advantage as it functions by sharing the load, reduced stress shielding, a decreased chance of refracture after implant removal, and autograft availability during reaming [7].

## Materials and methods

**Figure 1:**
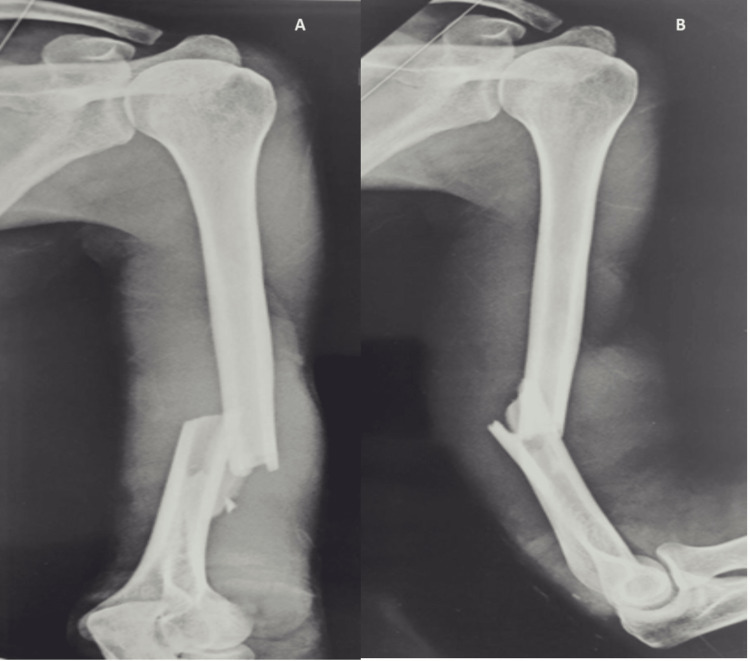
Radiograph of shaft humerus fracture (A) Anteroposterior view; (B) lateral view

This study, which is a retrospective observational study, was conducted in the orthopedic department of a tertiary trauma center in Mumbai. Records of individuals who had their shaft of humerus fractures (Figure 1) surgically fixed were examined between September 2021 and October 2022. Preoperative clinical assessment sheets, postoperative follow-up sheets, operative notes, anesthesia sheets, and preoperative and follow-up radiographs were analyzed.

The criteria used for selection were as follows: (1) humeral shaft fractures that needed surgery were managed with plating or interlock nailing techniques and (2) patients were at least 18 years old.

The following patients were excluded: (1) grade 3 compound fractures, (2) pathological fractures, (3) fractures within 4 cm of the proximal and distal end of the humerus, (4) patients aged less than 18 years, (5) patients with less than four months of follow-up, and (6) nonunions.

**Figure 2:**
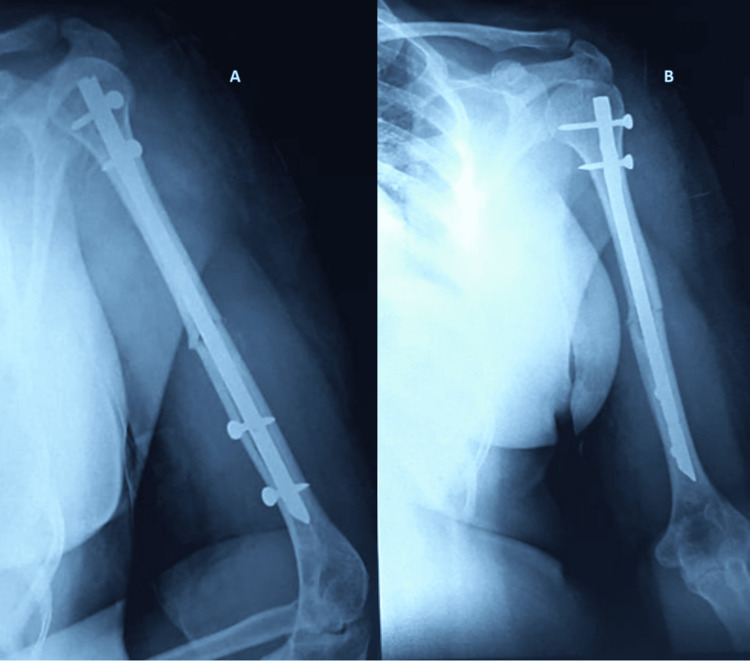
Postoperative radiographs of humerus shaft fracture fixed with intramedullary nailing (A) Lateral view; (B) anteroposterior view

**Figure 3:**
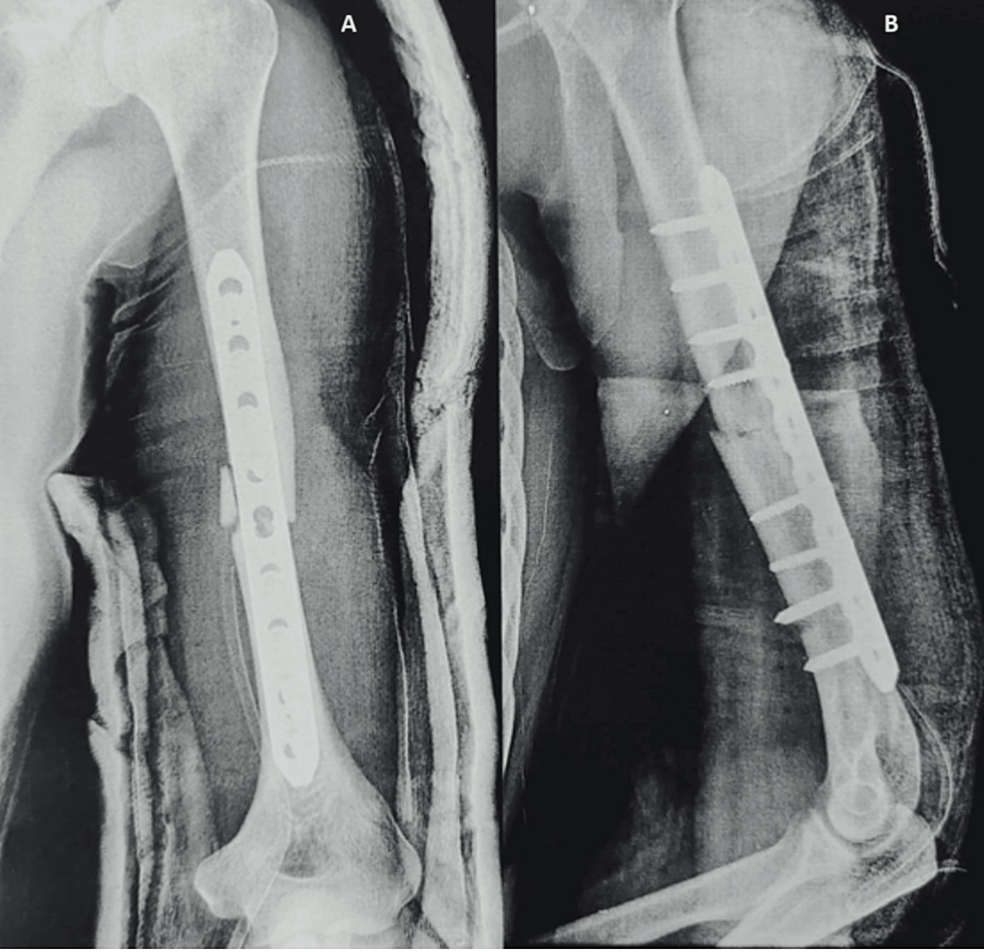
Postoperative radiographs of humerus shaft fracture fixed with dynamic compression plating (A) Lateral view; (B) anteroposterior view

We chose 10 patients who had intramedullary nailing (Figure 2) and 17 patients who had plating (Figure 3) for their shaft humerus fractures based on these criteria. The decision to go for a plating or a nailing surgery was left to the surgeons' choice and comfort of performing the particular procedure.

The same set of surgeons who were proficient in both surgical techniques operated on all the patients. A Russell-Taylor-type stainless steel intramedullary nail was inserted using an antegrade interlocking approach through the shoulder, and the rotator cuff was protected as much as possible. In every instance, proximal and distal locking and reaming were conducted.

Using the appropriate AO (Association for Osteosynthesis) principles, the plating group used a 3.5-mm or 4.5-mm stainless steel dynamic compression plate of different lengths, depending on the length and breadth of the bone. The posterior approach was used for this surgery. In each patient, eight to ten cortices were fixed, both proximally and distally to the fracture site. The slab was applied for protection until stitch removal.

At induction and for the following 48 hours, prophylactic antibiotics were given to all patients. After 48 hours, all patients went home, and the majority had their stitches removed after 14 days.

**Figure 4:**
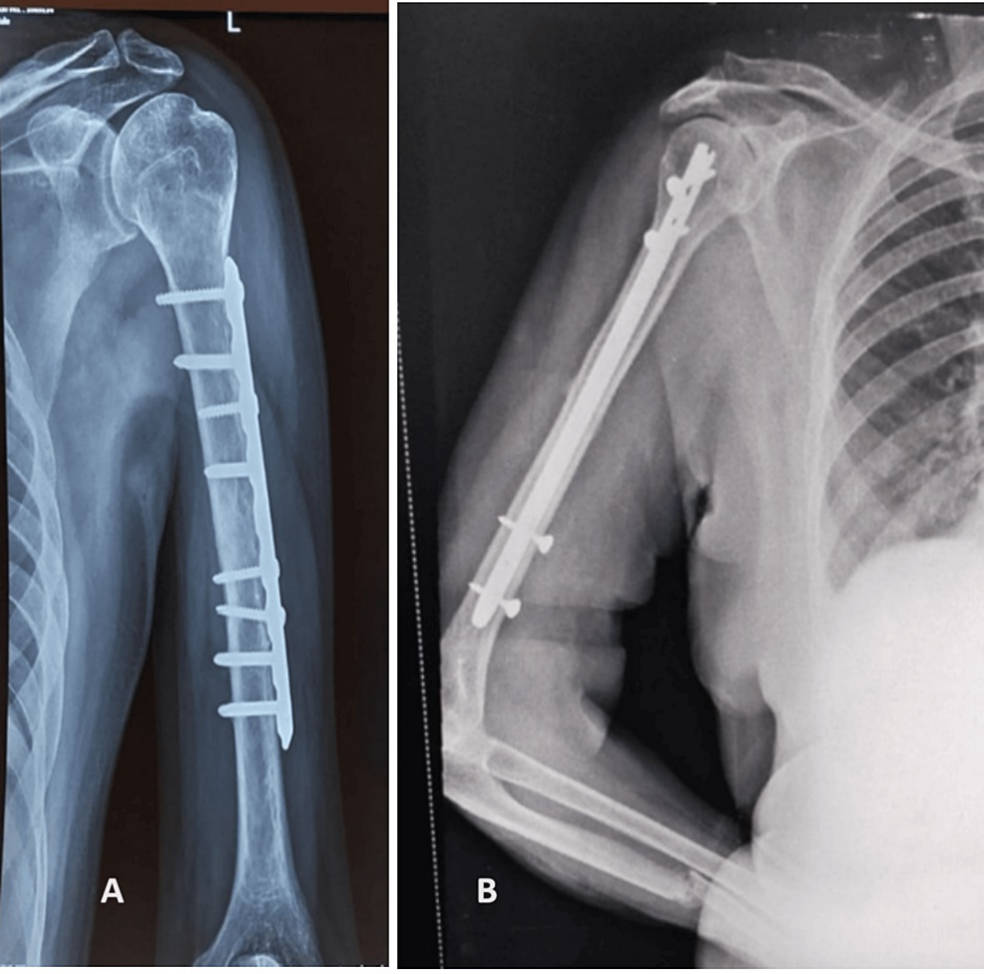
Six-month postoperative radiographs (A) Six-month follow-up radiograph showing a union at the fracture site after performing dynamic compression plating; (B) six-month follow-up radiograph showing union at the fracture site after performing interlock nailing

All patients were instructed to undergo shoulder and elbow physical therapy immediately after surgery, and radiographs were taken regularly during the follow-up period (Figure 4). The functional outcomes of the patients were analyzed using the Rodriguez-Merchan criteria [8] (Table 1). When any two different criteria fell into different groups, the outcome was classified using the lower category.

**Table 1:** Rodriguez-Merchan criteria for the functional assessment [8]

Rating	Elbow range of movement	Shoulder range of movement	Pain	Disability
Excellent	Extension 5°; flexion 130°	Full range of movement	None	None
Good	Extension 15°; flexion 120°	<10% loss of total range of movement	Occasional	Minimum
Fair	Extension 30°; flexion 110°	10–30% loss of total range of movement	With activity	Moderate
Poor	Extension 40°; flexion 90°	>30% loss of total range of movement	Variable	Severe

## Results

Demographics

**Figure 5:**
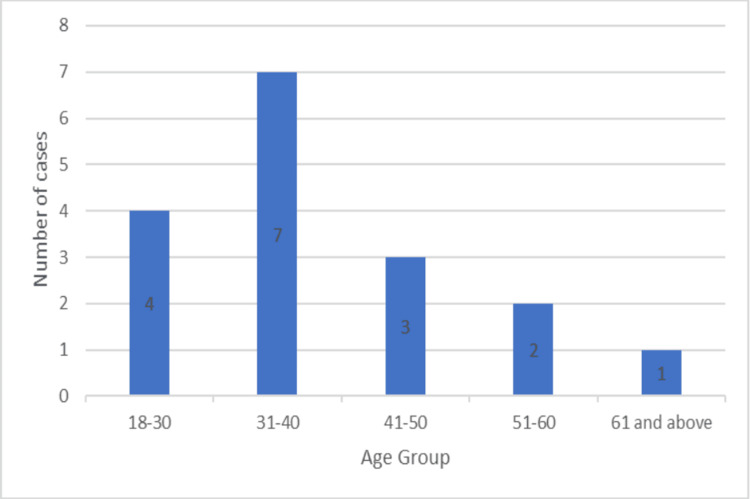
Age group of patients undergoing plating

**Figure 6:**
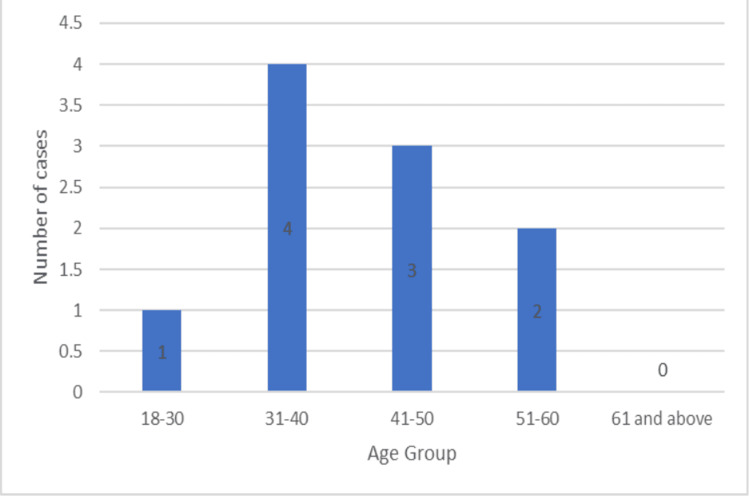
Age group of patients undergoing nailing

Approximately 17 patients underwent plating for their fractures. Of these, 13 (76.47%) were males, while four (23.53%) were females. Three (30%) and seven (70%) of the 10 patients who had intramedullary nailings were female. The patients that had plating had an average age of 39.1 ± 12.1, with the youngest being 25 years old, and the oldest being 72 (Figure 5). The average of the nailing group was 41.2 ± 9.37 years (Figure 6).

**Figure 7:**
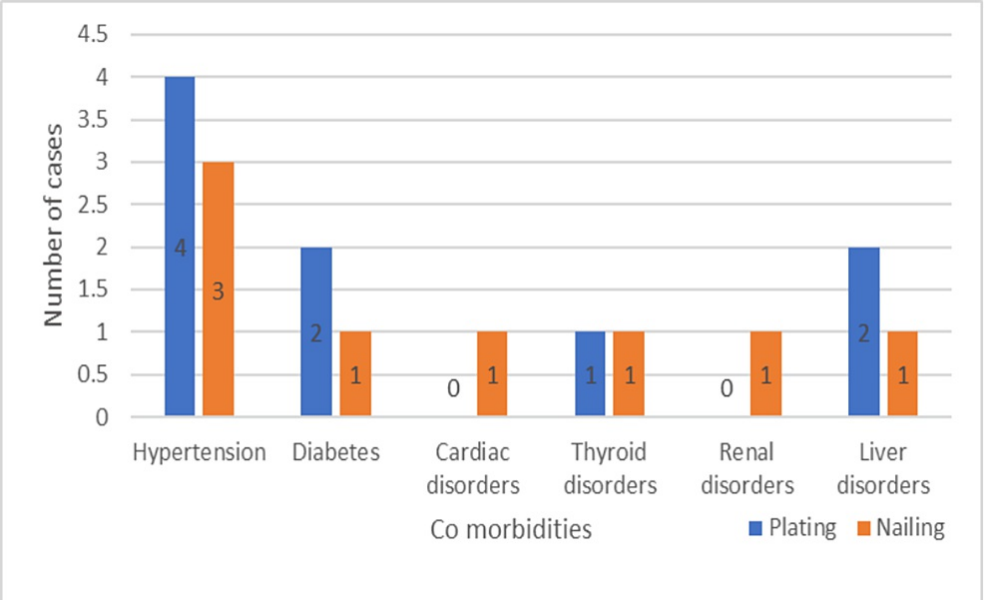
Comorbidities among the patients

**Figure 8:**
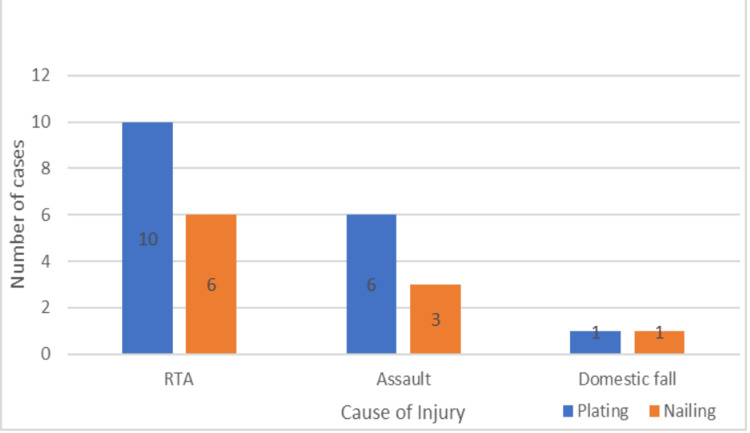
Cause of injury among the patients

**Figure 9:**
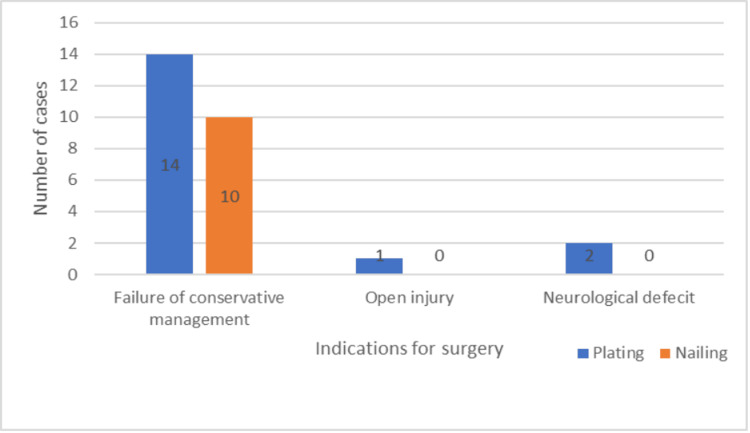
Indications for surgery

The patients in the two groups had comorbidities, among which hypertension was the most common one (Figure 7). In both groups, automobile accidents were the most frequent cause of injuries (Figure 8). However, the most frequent reason for surgery was a result of conservative therapy failing (Figure 9).

Operative results

The nailing group's operating time was 82.1 ± 7.61 minutes, which was much smaller (P value <0.05) than the plating group's 119.59 ± 10.16 minutes. 

Compared to the plating group, which experienced an intraoperative blood loss of 130.59 ± 11.44 mL, the nailing group's blood loss was considerably smaller (P value <0.05) at 71 ± 7.38 mL. All nailing procedures were closed under fluoroscopic guidance without opening the fracture site.

Functional results

**Figure 10:**
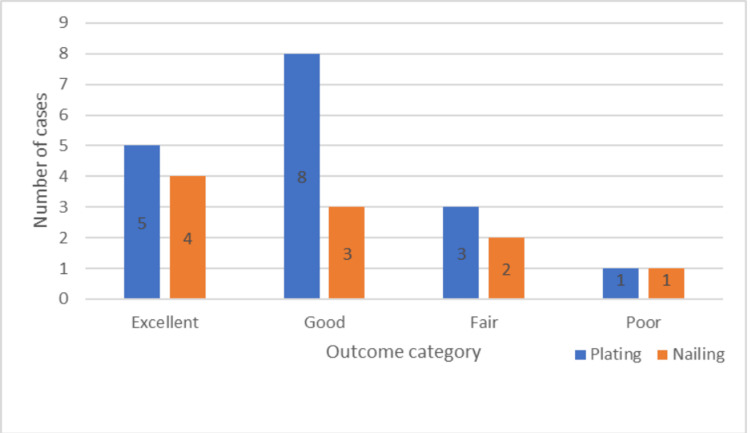
Postoperative functional results as per Rodriguez-Merchan criteria

Out of the 17 patients in the plating group, five (29.41%) had excellent results, while eight (47.06%) had good results. In the case of the nailing group, four (40%) had excellent results, while three (30%) had good results. The two groups did not differ statistically from one another (Figure 10).

Complications

**Figure 11:**
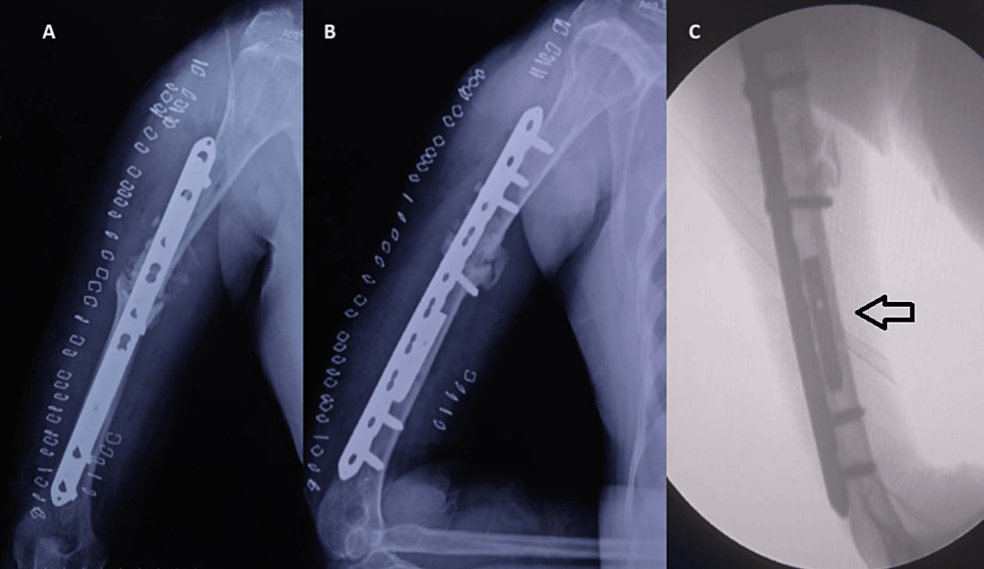
Postoperative radiograph of failure of Intramedullary nailing managed with dynamic compression plating and bone grafting (A) Lateral view; (B) anteroposterior view; (C) fluoroscopic intraoperative image with an arrow showing a retained piece of Intramedullary nail within the humeral shaft canal

Preoperative neurological injury was seen in two (11.76 %) of the patients who underwent plating. No preoperative neurological deficit was seen in the nailing group. All of the patients with preoperative radial nerve injury recovered postoperatively after fracture fixation, which indicates a neuropraxia type of injury. Of the patients in the plating group, two (11.76%) experienced postoperative radial nerve palsy. No postoperative neurological injury was seen in the nailing group. Both cases recovered within three months of surgery, and there was no need to explore the radial nerve. Three (17.65%) of the plating group patients had surgical site infections. One was a severe infection that was treated with washout and ongoing antibiotic therapy before progressing to union. Two cases were superficial surgical site infections that were managed with the use of antibiotics. There were no cases of surgical site infection in the nailing group, and two (20%) of the patients in the nailing group had rotator cuff impingement because of the protruding implant. It was managed with implant removal as soon as union was achieved. Three patients each in the nailing (30%) and plating (17.65%) went into a delayed union (union taking greater than 16 weeks). One case each in the nailing (10%) and plating (5.88%) went into nonunion. They were managed with repeat plating with bone grafting and eventually went on to unite. In one case of intramedullary nailing, there was a breakage of the nail. This was resolved by plating with bone grafting and removing the nails. The distal part of the broken implant was not removed (Figure 11).

## Discussion

Most of the patients in our study were males in both the plating and the nailing groups. In the Changulani et al. study in the dynamic compression plating group, there were 19 men (79.2%) and five females (20.8%), compared to 20 males (86.9%) and three females (13%) in the intramedullary nailing group [6]. In the Kurup et al. study, the percentage of males ranged from 61% to 83% [4]. This is similar to our study. Furthermore, the mean age of the patients in our investigation was 41.2 ± 9.37 years for the nailing group and 39.1 ± 12.1 years for the plating group. The age categories with the highest number of patients in the study were 31-40 and 41-50. The study by Beeres et al. found that the median age was 42 (ranged 16-88) years. The plate fixation group's age was 41 years, while the nail fixation group's age was 42 years [9].

In our study, the majority of shaft humerus fractures were caused by traffic accidents; 58.82% of the plating group and 60% of the nailing group experienced these injuries. In the study by Changulani et al., road traffic accidents were the most common cause of humerus shaft fractures in both plating (73%) and nailing (66.6%) groups [6]. Similar results were obtained in other studies and matched our study observations [10-13]. Failure of conservative management was the major reason for operative treatment in our study, with 82.35% in the plating group and 100% in the nailing group. This is in accordance with most studies [4,6,10,14].

When it comes to surgically fixing shaft humerus fractures, plating and intramedullary nail fixation are the most popular techniques. In community, segmental, and pathological fractures, interlocking nailing is favored; while when radial nerve exploration is being considered, plating may be the preferred option [10,14-16]. The external fixation approach may be employed for open wounds and is less common in the treatment of humeral shaft fractures [13].

The mean operative time in the plating group was 119.59 ± 10.16 mins, while in the nailing group, it was 82.1 ± 7.61 mins. The mean blood loss in the plating group was 130.59 ± 11.44 ml, while in the nailing group, it was 71 ± 7.38 ml. Both of them were significantly lesser in the nailing group [4,9]. Only in the study by McCormack et al., no significant difference was seen between the two groups [14]. Most of the patients in the plating and nailing group had excellent to good results postoperatively as per the Rodriguez-Merchan criteria [8,14,17].

There have been general concerns raised in the case of dynamic compression plating regarding infection, nonunion, and radial nerve palsy [4,6,8-10]. Comparably, in our investigation, the plating group experienced three instances of infection, while the nailing group experienced no surgical site infections. In the study by Bhandari et al., no statistical significance was seen between the two groups [18]. Restriction of shoulder motion has been raised as an issue with intramedullary humeral nail use [18-19]. Shoulder dysfunction with the antegrade interlocking nails may be caused by impingement from proximal nail migration, rotator cuff damage, adhesive capsulitis, or an unidentified etiology [14,18-19]. About 20% of the cases in the nailing group had shoulder movement restriction with rotator cuff impingement, which was managed by early implant removal after fracture union. In surgically treated humeral shaft fractures, the nonunion rate ranges from 0 to 8%, with comparable rates in the plating and nailing groups [9,20]. In the Changulani et al. study, nonunion happened in three (14.3%) of the intramedullary nailing compared to three (12%) of the dynamic compression plating group [6]. One case each in the nailing (10%) and plating (5.88%) went into nonunion in our study, which is similar to most literature on nonunion rates. Both cases were managed with bone grafting. Approximately 11.76% of the patients in the plating group had radial nerve palsy, which there was no case in the case of the nailing group. In the research by Singisetti et al., postoperative radial nerve palsy was observed in one instance (6.25%) in the plating group, but not in any of the nailing group [10]. Only one out of 24 patients had an iatrogenic nerve I jury after plating in the Changulani et al. study [6].

Both nailing and plating had similar functional results as per our study, similar results were obtained in the Singisetti et al. study [10]. Both surgical time and surgical blood loss were significantly lesser in the nailing group as compared to the plating group. Similar results were seen in the meta-analysis done by Beeres et al. [9].

Limitations

This study had a small sample size and was conducted retrospectively in a single center. A multicenter prospective study with a larger sample size is our recommendation for further analysis of these two surgical modalities.

## Conclusions

In conclusion, both intramedullary nailing and dynamic compression plating are good options for surgical fixation of the shaft of humerus fractures. Intramedullary nailing is a faster procedure with lesser blood loss. However, it is associated with complications like rotator cuff impingement. Radial nerve injury is more common with dynamic compression plating surgeries. We can conclude that each surgery must be individualized as per the patient and surgeon's surgical competence with the technique to achieve the best possible result.
